# Breeding Biology of the Twite *Linaria flavirostris* in the North-Eastern Qinghai–Tibet Plateau, with Special Reference to Life-History Variation Across Latitudes and Altitudes

**DOI:** 10.3390/ani16091395

**Published:** 2026-05-02

**Authors:** Shuai Yan, Bowen Zhang, Shaobin Li

**Affiliations:** 1 Hubei Provincial Key Laboratory for Protection and Application of Special Plant Germplasm in Wuling Area of China, College of Life Sciences, South-Central Minzu University, Wuhan 430074, China; shuai_yan@yeah.net; 2 College of Life Sciences, Yangtze University, Jingzhou 434025, China; bowen_zhg@126.com

**Keywords:** Fringillidae, *Linaria*, high-altitude region, riparian shrubland, reproductive biology, growth curve

## Abstract

In 2024 and 2025, researchers investigated the breeding ecology of the Twite *Linaria flavirostris* in riparian shrubland habitats at an elevation of 3400 m in the northeastern Qinghai–Tibet Plateau. This species lays eggs from late June to mid-July, capitalizing on the region’s brief warm season. Nests are typically open-cup structures built in *Hippophae* spp. shrubs. The population predominantly exhibits monogamous mating, with a mean clutch size of 4.7 ± 0.49 (3~5). Incubation is performed solely by the female and lasts 11.52 ± 1.65 days. Both parents provision the nestlings, and the nestling period lasts 12.43 ± 2.39 days. Morphological measurements of nestling body mass and external organs all fit well to the Logistic growth curve equation. By fledging, tarsus length and bill length reach over 90% of adult values, conferring substantial terrestrial mobility. However, flight-related feathers, primaries and rectrices, remain markedly underdeveloped compared to adults, resulting in extremely poor flight capability; further post-fledging development is thus required. Based on reproductive outcomes from this single breeding season, a total of 121 eggs were laid, of which 81 successfully hatched, and ultimately 79 fledglings survived to leave the nest. The overall hatching success was 66.94%, fledging success (among hatchlings) was 97.53%, and overall offspring survival (from eggs to fledglings) was 65.29%. The apparent nesting success rate was 76.0%, based on a total of 50 nests monitored over two years. Daily nest survival rates were estimated using Mayfield’s method and program MARK, resulting in nest success probabilities of 0.587 and 0.219, respectively. Comparing populations across different geographic regions, the results indicate that Twites breeding in environments with higher levels of environmental stress produce smaller clutch sizes and larger eggs, and exhibit a prolonged nestling period. This life-history strategy likely represents an evolutionary adaptation to spatially variable environmental conditions.

## Simple Summary

The Qinghai–Tibet Plateau (QTP), despite its harsh environmental conditions, is one of the global hotspots richest in biodiversity. The Twite (*Linaria flavirostris*) thrives and breed successfully in this region. It serves as a good model for studying avian adaptation to high-altitude, cold, and hypoxic environments, and acts as an indicator species for assessing the ecological health of the QTP ecosystem. While extensive research on this species exists internationally, studies conducted at high altitudes are still relatively scarce. Through two consecutive breeding seasons of field surveys and observations on the northeastern QTP, we monitored the breeding process of a local population of the Twite. We compiled a comprehensive table of breeding parameters for this population and fitted Logistic growth curves to analyze the development of nestlings. By integrating and comparing our findings with previously published reproductive biology data, we discovered that populations inhabiting more extreme environments tend to produce smaller clutch sizes but lay larger and heavier eggs, and invest more time and energy in parental care for their fewer offspring. Our study demonstrates that this species effectively adapts to the extreme cold of the QTP through the optimization of various reproductive strategies. These findings elucidate the reproductive characteristics of the Twite in high-altitude cold environments and reveal its ecological adaptive mechanisms in response to increasingly severe conditions. This research provides empirical evidence and novel data supporting changes in avian life-history strategies under high-altitude cold stress. Consequently, it offers valuable references for environmental conservation and biodiversity protection efforts within the QTP ecosystem.

## 1. Introduction

Avian reproductive biology, a key subdiscipline of animal ecology, focuses on birds’ reproductive behaviors, physiological mechanisms, and ecological strategies, examining their interactions with the environment. This field is crucial for understanding avian population persistence and ecosystem stability. Its core encompasses a suite of behaviors—including mate choice, nest building, egg laying, and chick rearing—as well as the adaptive relationships between species-specific reproductive traits and environmental conditions [1,2]. The Qinghai–Tibet Plateau (QTP), known as the “Roof of the World,” is characterized by extreme environmental conditions—including high altitude, low temperatures, hypoxia, and intense ultraviolet radiation—yet it harbors an exceptionally rich avian fauna, with over 700 recorded bird species, accounting for more than 50% of China’s total bird diversity [3]. This unique pattern of biodiversity distribution has made it a focal region for global research in biogeography, evolutionary ecology, and related fields, offering irreplaceable value in elucidating the mechanisms underlying the assembly and maintenance of biological communities in extreme environments [4].

To cope with the dual survival pressures imposed by extreme environmental stressors such as low temperature, hypoxia, and intense solar radiation and the highly spatiotemporally fragmented and overall scarce availability of food resources in alpine regions, high-altitude birds have evolved a suite of reproductive ecological strategies finely tuned to their environment over long-term evolutionary processes. For instance, they concentrate egg-laying and chick-rearing within the narrow window of the warm season to avoid risks associated with cold temperatures and food scarcity [5,6]; reduce clutch size to alleviate energetic demands during a single reproductive attempt [7,8]; and intensify parental care by extending the nestling period and increasing feeding frequency to ensure adequate nutritional provisioning for offspring [9,10].

The Twite *Linaria flavirostris* is a small passerine finch characterized by a yellow bill, predominantly brownish plumage, and a pale rufous rump. It is omnivorous, highly gregarious, and exhibits no pronounced sexual dimorphism. This species has a broad distribution across the boreal and temperate regions of Europe and northern to central Asia, primarily inhabiting high-altitude areas. Some populations undertake short-distance or altitudinal migrations in response to seasonal changes [11,12,13,14]. As a key component of alpine food webs and a species of significant ecological value in China, the Twite serves as an indicator of high-altitude habitat health, contributes to ecosystem stability through seed dispersal, and acts as a crucial monitor for environmental changes and biodiversity status.

According to current authoritative records, the IUCN Red List status of Linaria flavirostris is assessed as Least Concern (LC). This assessment indicates that the species currently maintains a relatively stable population trend and a wide distribution range, facing no imminent risk of extinction. However, given the significant decline in population numbers observed in specific regions (e.g., the United Kingdom), the dynamics of local populations warrant continued monitoring [4]. In China, where the species has a relatively limited distribution primarily restricted to high-altitude regions such as the Qinghai–Tibet Plateau, it is included in the List of Terrestrial Wildlife with Important Ecological, Scientific, and Social Values (the “Three-Native” list) and is thus afforded legal protection [15].

With advances in disciplinary understanding and technological progress, the taxonomic placement of the Twite has undergone successive revisions—initially classified within the genus *Acanthis*, later transferred to *Carduelis*, and ultimately reassigned to *Linaria*, which reflects an ongoing, dynamic refinement in our understanding of its phylogenetic relationships. Meanwhile, systematic research on this species encompassing its reproductive behavioral strategies, the evolution of migratory patterns, and population dynamics, has already accumulated extensive research findings [16,17,18,19,20,21,22,23].

This study focuses on a population of *Linaria flavirostris* inhabiting riparian shrubland at an elevation of 3400 m in northeastern Qinghai Province. We describe its reproductive parameters and ecological adaptations during the breeding season, with the aim of identifying the scientific gap of this species in reproduction in extreme alpine environments and enriching basic data on both reproductive ecology and nestling growth for this species in high-altitude, extreme habitats. Additionally, by synthesizing breeding-related information from observational records of other populations—both domestic and international—we conduct a cross-population comparative analysis to further explore the co-evolutionary dynamics between the species’ life-history strategies and alpine environmental factors. This research provides a scientific basis for the conservation of Twite populations and for advancing our understanding of their adaptive mechanisms in harsh high-elevation environments.

## 2. Materials and Methods

### 2.1. Location of the Study

**Figure 1:**
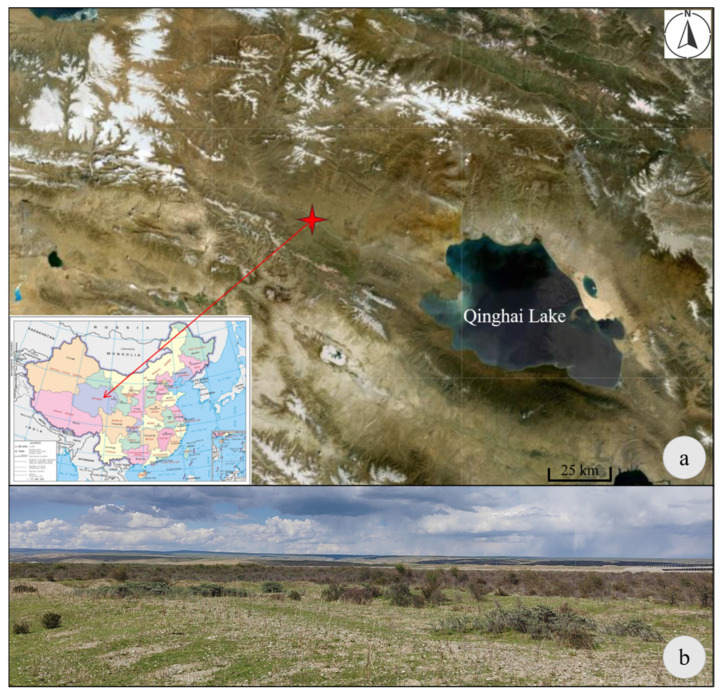
(**a**) Geographical location and (**b**) environmental characteristics of the research site.

Tianjun County is located in the northeastern part of the Qinghai–Tibet Plateau and belongs to the Haixi Mongol and Tibetan Autonomous Prefecture of Qinghai Province, China (Figure 1). The county’s terrain is predominantly characterized by mountains and alpine meadows, with an average elevation exceeding 4000 m above sea level. It experiences a high-altitude cold climate, featuring long, severely cold winters and short, cool summers. The annual mean temperature is approximately –1.5 °C, with an extreme minimum temperature of –35.8 °C. Precipitation is relatively low, averaging about 360 mm annually, and is unevenly distributed throughout the year; rainfall coincides with the warmer season, resulting in distinct wet and dry periods. The region is frequently subjected to strong winds and dust storms, with an average of 97 days per year experiencing gale-force winds. Solar radiation is abundant: the annual sunshine percentage reaches approximately 70%, and the annual solar radiation totals about 612 kJ/cm^2^—significantly higher than that observed in low-elevation regions.

The primary study site was located in riparian shrublands near the Buha River Bridge in Tianjun County (37.31° N, 99.01° E; 3400 m a.s.l.). Dominant local vegetation includes *Hippophae* spp., *Myricaria* spp., and *Tamarix* spp., which are the principal shrub species selected by Twite for nesting. Additional plant materials used in nest construction include *Festuca rubra*, *Calamagrostis epigeios*, and *Clematis tangutica*.

### 2.2. Nest Localization

Bird nests were located using a combination of systematic ground searches, tracking bird vocalizations, following adult birds carrying nesting material, and telescope-based observation. Nests were considered active based on the presence of eggs or nestlings and the condition of nesting materials. The species can be identified based on the characteristics of the eggs in the nest and the returning or alerting adult birds. All the nests are numbered according to the species and the discovery sequence, and then their exact locations are recorded using software tools such as GPS (mobile application gpstool v2.9.6).

### 2.3. Data Collection

**Figure 2:**
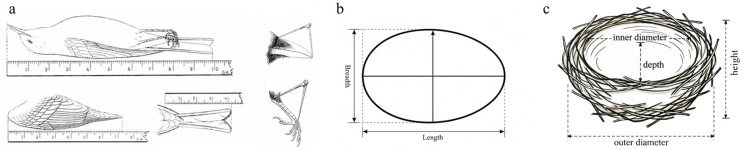
Measurements of morphometric parameters of (**a**) avian individuals [24], (**b**) eggs and (**c**) nests.

Active nests were visited at regular intervals (1 or 2 days) to monitor reproductive status and destiny. For newly laid eggs, we recorded their length, breadth (Figure 2b), and fresh mass. Nestlings were measured during every visit for six morphological traits (Figure 2a), body length (BL), wing length (WL), tail length (TL), tarsus length (TSL), bill length (BIL), and body mass (BM), and then marked with colored waterproof felt-tipped pens. During the breeding season, mist nets were set up near active nests to capture attending adults. Sex was determined based on the presence of a brood patch (in females) or a developed cloacal protuberance (in males). In addition to the six nestling measurements, adult birds were measured for five additional traits: bill width (BIW), bill depth (BID), head-bill length (HBL), head width (HW), and head height (HH). All morphological traits were measured to the nearest 0.1 mm (lengths) and 0.1 g (mass) using electronic digital calipers and a portable electronic balance, respectively. After being banded with unique combinations of plastic color rings, birds were released immediately.

Once all nestlings in a nest fledged, the nest structure was thoroughly measured (Figure 2c) using a tape measure (to the nearest 0.1 cm) and weighed using an electronic balance (to the nearest 0.1 g) and documented. These parameters included nest height above ground, distance to the tree top, inner and outer diameters, nest depth, and nest height.

### 2.4. Data Processing

Field investigations were conducted from June to August in both 2024 and 2025. To better characterize the population status of the species at the study site during the non-breeding season, a field survey was conducted again in November 2025.

In total, 50 Twite nests were located during this study (24 nests in 2024 and 26 nests in 2025). Not all bird nests are located before the breeding begins; some nests are already in different stages of reproduction (incubating or nestling periods) when discovered. In addition, the breeding process of birds is a continuous one; therefore, the dates of the first egg laid in certain breeding nests, that is, the dates of clutch initiation, were determined by backdating, using the means of nesting parameters established based on closely monitored nests.

Statistical analyses and function fitting were mainly performed using SPSS 26.0. To avoid some inconvenience, some dates are expressed in Julian date (JD). All statistical values are given as mean ± SD.

## 3. Results

### 3.1. Social Organizations and Behaviors

Field surveys conducted during the summer and winter of 2025 revealed that Twite were present in the study area year round. Outside the breeding season, individuals typically form flocks ranging in size from a dozen to over one hundred birds, occasionally forming temporary and loose mixed-species foraging groups with other birds, such as *Passer montanus*. Winter flocks are smaller, with around a dozen individuals, while some individuals undertake altitudinal migration, moving to lower-elevation areas to overwinter. During the breeding season, they occur in pairs and predominantly exhibit monogamous mating behavior. This species exhibits weak territoriality, allowing incubating females to be captured by hand.

### 3.2. Nest Building and Nest-Site Selection

Following successful mating, nest construction is carried out solely by the female. The sexes were distinguished based on post-capture examination and plumage differences, with males exhibiting a pinkish rump while females were generally duller. The nest is an open, cup-shaped structure. Its primary nesting materials, from the outer to the inner layers, consist of twigs, various grasses, plants’ stylar hairs, and animal hair (yaks and sheep). A few nests also contain small amounts of artificial materials, such as plastic and sponge. Nest construction typically takes about 4 to 7 days.

The characteristics of nest-site parameters are summarized in the discussion section. The outer diameter of 50 nests was 9.9 ± 0.8 cm (8.3~11.8), the inside diameter was 5.8 ± 0.8 cm (4.0~7.5), the depth of the nest cup was 3.7 ± 0.6 cm (2.0–5.5), and the dry mass of 50 nests averaged 17.5 ± 5.3 g (9.4~32.7), with grasses accounting for approximately 70%.

The study site is relatively barren and supports a limited diversity of plant species. The vegetation is dominated by shrubs approximately 1.5 m in height, with the remainder consisting of herbaceous plants. However, three tree species are available for Twite to use as nesting substrates. However, 96.0% of all observed nests were built in sea buckthorn (*Hippophae* spp.), with only one nest each found in *Potentilla fruticosa* and *Myricaria* spp. A chi-square test (χ^2^ = 13.184, *p* = 0.004) confirmed that Twite exhibits a significant preference for nesting in sea buckthorn. This is primarily because the species possesses a distinct, upright main stem; the branching points provide stable support for nest construction, while the hard, dark thorns at the branch tips effectively deter predators and other animals. Notably, some trees were associated with *Clematis tangutica*, a herbaceous vine that forms achenes with numerous persistent styles after flowering, which enhances nest concealment and facilitates access to nesting materials.

The mean height of nesting trees was 173.5 ± 33.6 cm (58.5~248.0). Nests were typically located at a height of 127.2 ± 31.5 cm (31.0~185.0) above the ground and 39.8 ± 16.7 cm (16.0~84.0) below the tree canopy. Nest distribution aligned with the orientation of the river channel, with nests present in shrubs on both banks. The perpendicular distance from nests to the river channel was approximately 100 m.

### 3.3. Egg Laying and Breeding Season

**Figure 3:**
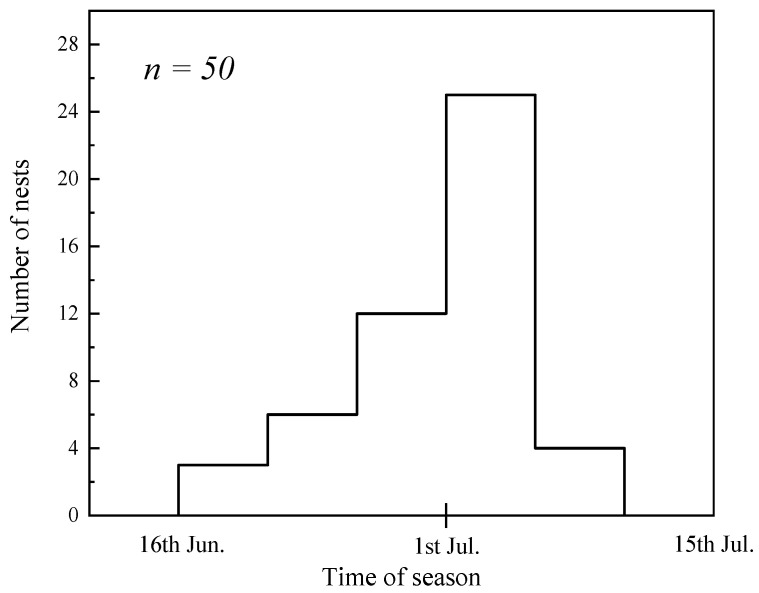
Distribution of clutches according to the laying date of first egg.

The results indicate that the breeding period of this Twite population spans from late June to early August (JD 173–218, Figure 3), lasting approximately 45 days in total. The peak breeding activity occurs around the start of July. The earliest recorded initiation date was 22 June (JD 173), while the latest termination date was 6 August (JD 218).

The breeding period, defined as the interval from the laying of the first egg to the fledging of the last nestling, averaged 28.1 ± 4.3 days, comprising an egg-laying period of 4.6 ± 0.5 days, an incubation period of 11.5 ± 1.7 days, and a nestling period of 12.4 ± 2.4 days.

### 3.4. Egg and Clutch Size

Egg-laying occurred at a rate of one egg per day. Eggs of this Twite population were distinctly ovoid, ellipsoidal with one end noticeably more blunt than the other, which may help shorten the incubation period [25], and have a rough surface texture. They were predominantly creamy white, adorned with irregularly sized and unevenly distributed black spots and pale reddish-brown blotches, which were concentrated toward the blunt pole. Based on measurements of 137 eggs, mean egg length was 17.3 ± 0.7 mm, and mean breadth was 12.6 ± 0.4 mm. The calculated mean egg volume was 1411.2 ± 132.6 mm^3^ [26], and mean egg fresh mass was 1.4 ± 0.18 g. The average clutch size was 4.7 ± 0.5 (3~5), with five-egg clutches being most common (65.38% of all clutches).

### 3.5. Incubation and Care of Nestlings

Incubation was performed exclusively by females, with most initiating this behavior upon laying the penultimate egg of the clutch. The incubation period, defined as the time elapsed from the last egg laid to the first egg hatched, was 11.5 ± 1.6 days (10~14).

A portion of the female’s energy requirements is dependent on the male’s courtship feeding, whereby the male brings back foraged food to the nest and feeds it to the female; meanwhile, the female also leaves the nest briefly to forage on her own during incubation breaks. Both parents provisioned the nestlings, and females brooded the nest in the early nestling period. However, as nestlings developed, both the frequency and duration of brooding behavior gradually decreased.

Upon hatching, nestlings are incapable of foraging independently and rely entirely on parental provisioning. Adult birds make frequent trips to the nest, delivering food directly to the chicks. They regurgitate partially digested food, from the crop, and transfer it beak-to-beak into the nestlings’ mouths. The diet provided is omnivorous, consisting primarily of *Diptera* (e.g., mosquitoes) and other insects, supplemented with plant seeds. When detecting the return of a parent, nestlings exhibit pronounced food-begging behavior: they rapidly assume an upright posture, stretch their necks, gape widely, vibrate their bodies, and emit persistent vocalizations to compete for food delivery [27].

### 3.6. Reproductive Success

In 2025, among the 26 breeding nests, a total of 121 eggs were recorded. Of these, 100 eggs reached the hatching stage, 81 successfully hatched into chicks, and ultimately 79 nestlings fledged. For this population in 2025, the overall hatching success rate was 66.94%, the fledging rate (of hatched chicks) was 97.53%, and the overall offspring survival rate (from egg to fledgling) was 65.29%.

Among the 50 breeding nests found over two years, 38 were successful (that is, at least one chick fledged), resulting in an average nest success rate of 76%. Breeding failures were due to nest loss caused by nest destruction (*n* = 5, 10%) or nest abandonment (*n* = 7, 14%). Including the nest-building period, the entire nesting period lasts approximately 33 days. The total nest survival probability estimated by Mayfield’s method [28] was 0.587. The daily nest survival rate (DSR) estimated using program MARK [29] was 0.955; consequently, the probability of nest success was 0.218. The incorporation of these two approaches provides additional alternatives for the characterization of this indicator.

### 3.7. Growth of Nestlings

#### 3.7.1. Logistic Growth Curves

**Figure 4:**
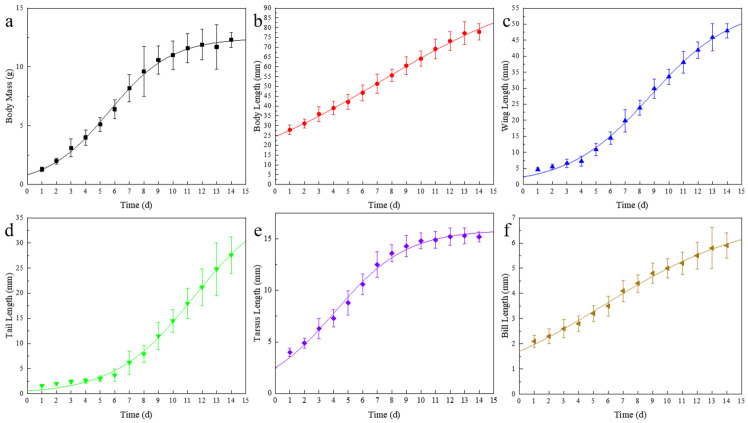
Logistic growth curve fitting diagrams during the nestling period: (**a**) Body mass; (**b**) Body length; (**c**) Wing length; (**d**) Tail length; (**e**) Tarsus length; (**f**) Bill length.

We dynamically characterize the nonlinear growth and development of nestlings (58 individuals from 17 nests) by continuously measuring six morphological traits and fitting Logistic growth curves using nonlinear regression, thereby analyzing their associated biological characteristics [30,31,32,33]. The fitting results are as follows (Table 1 and Figure 4). In the expression, the inflection point occurs at Y = A/2, where the very slope represents the maximum growth rate of nestlings for this trait.

#### 3.7.2. Differences Between Males and Females

All captured adult individuals were grouped by sex. Due to unequal and small sample sizes in both groups, Mann–Whitney U tests were conducted on all eleven phenotypic traits. The results (Table 2) show that females exhibited smaller values than males across all traits; however, most of these differences did not reach statistical significance. Only wing length (*p* = 0.005) and tail length (*p* = 0.008) exhibited statistically significant differences. In the subsequent analysis of differences between nestlings and adults, the aforementioned two indicators in nestlings are far below adult levels. Moreover, sex identification in nestlings is extremely difficult. Therefore, we did not consider sex differences in the growth of the nestlings at this stage.

#### 3.7.3. Differences Between Adults and Juveniles

After a nestling period of approximately 12 days, the fledglings become fully feathered and are ready to leave the nest. By this stage, their body mass and tarsus length have already reached over 90% of the adult average, closely approaching adult levels and conferring substantial terrestrial mobility. However, wing length and tail length, which are directly associated with flight capability, still lag considerably behind those of adults. Consequently, newly fledged juveniles exhibit very poor flight performance, with both flight distance and altitude typically less than 1 m. A detailed comparison between pre-fledging nestlings and adults is provided in the table below (Table 3).

**Figure 5:**
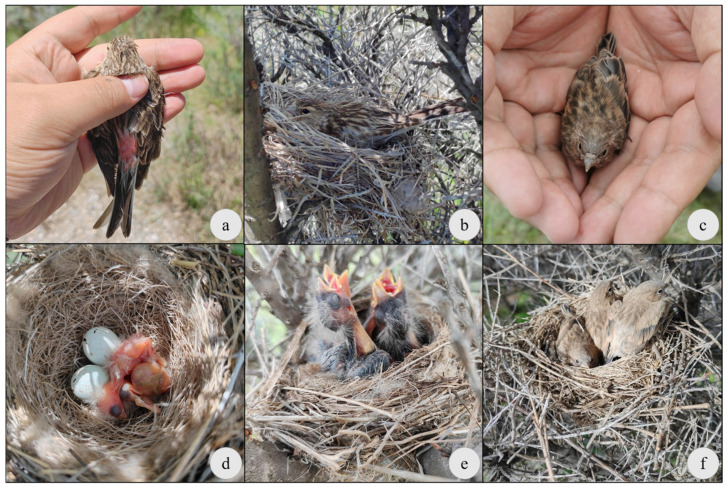
Pictures of Twites: (**a**) Male with characteristic pink waist. (**b**) Female sitting in the nest. (**c**) A fledgling that has just left the nest. (**d**) The eggs and the newly hatched hatchlings. (**e**) Nestlings begging for food. (**f**) Nestlings about to leave the nest.

By combining the growth curves of nestlings with the proportion of adult values they attain, we found that different external traits reach their peak growth rates at different times. The sequence, from earliest to latest, is tarsus length (day 3.5) < bill length (day 5.5) < body mass (day 5.6) < body length (day 7.3) < wing length (day 8.5) < tail length (day 11.3). Traits related to basic locomotor ability, represented by tarsus length, reach their maximum growth rate first and achieve near-adult size well before fledging. In contrast, traits closely tied to flight ability, namely the primary flight feathers on the wings, which generate lift, and the tail feathers, which control direction and balance, do not fully mature before fledging and require additional post-fledging development to function properly. In short, nestlings prioritize the development of basic movement abilities first, and flight-related features develop last. This aligns perfectly with the typical growth pattern of altricial nestlings, serving as an adaptive strategy for such harsh alpine environments. Photographs of *Linaria flavirostris* at various developmental stages are presented in Figure 5.

## 4. Discussion

### 4.1. Metrics of Reproductive Success

According to statistical results, the breeding success rate of Twite *Linaria flavirostris*, appears unusually high compared to other open-nesting bird species, whose breeding success rates generally do not exceed 50% [34,35,36]. Several factors may contribute to this outcome:

Firstly, low habitat disturbance. The primary nesting trees, sea buckthorn, are densely covered with thorns, effectively deterring predators and intruders. There is minimal activity from large animals within the habitats; occasional sightings of deer and stray livestock like horses have been noted. Four-fifths of the habitats are semi-protected, and during the total survey period of approximately 120 days, human activities were observed only twice without any grazing activities. The remaining one-fifth near a bridge has a few Tibetan residents living there temporarily, but their range of activity does not overlap significantly with the nesting areas of Twite. Due to religious beliefs, discovered nests are left largely undisturbed. Human presence (local inhabitants or researchers) might also deter potential predators in the environment, indirectly reducing nest predation. Predation, however, is the primary cause of nest failure, accounting for approximately 80% of such cases [37].

Secondly, “survivorship bias.” All nests identified for the first time were in the egg-laying, incubation, or nestling stage. Regardless of their future destiny, these nests had successfully passed the initial “nest-building” phase. Nests that failed to be built or existed for a short period of time would not be found and recorded, leading to an underrepresentation of failures and a smaller sample size overall, thus inflating the apparent breeding success rate. For this reason, we also employed two additional methods (program MARK and Mayfield’s method) to estimate nest success, which can, to some extent, help refine or correct the calculated results. In fact, they also worked.

### 4.2. Intraspecific Population Variation

As a widely distributed species, the Twite exhibits certain differences among populations from different geographic regions. Here, we compile and comparatively analyze existing research findings on the reproductive biology of this species across its range.

#### 4.2.1. Reproductive Parameters Across Populations

The reproductive parameters of different populations breeding in various habitats are summarized below (Table 4). Reproductive data were sourced from studies conducted in northern Scotland [20], the southern Pennines of England [38], southern and northern Tibet [39], central Tibet [40], northern Qinghai [41], and the present study.

**Figure 6:**
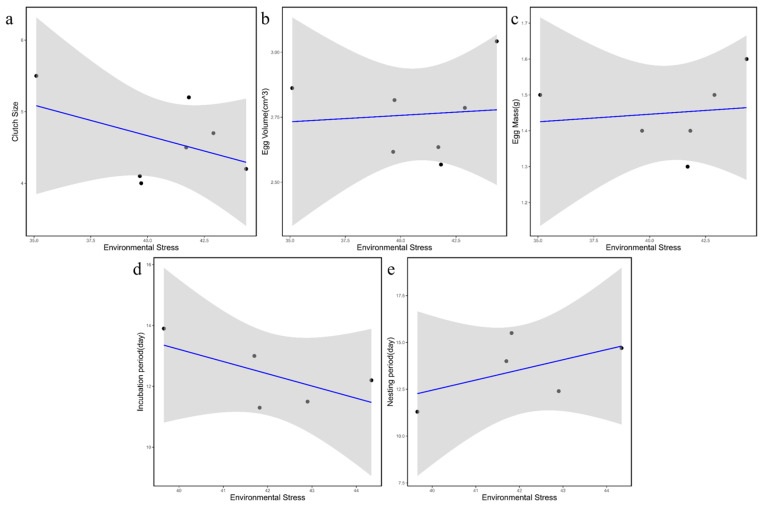
Correlation analysis of five sets of reproductive data with environmental stress: (**a**) Clutch size; (**b**) Egg volume; (**c**) Egg mass; (**d**) Incubation period; (**e**) Nesting period. The gray area represents the 95% confidence interval. The dots represent the respective indices of each habitat; the blue line indicates the regression line, while the gray area represents the 95% confidence interval.

High latitudes and high altitudes both result in reduced average temperatures, which exacerbates the harshness of avian habitats. For every 1° increase in latitude or every 100 m rise in elevation, the temperature decreases by approximately 0.6 °C. Therefore, we used the idealized condition of 0° latitude and 0 m elevation as a baseline. We defined environmental stress as a simple quantitative measure based on the theoretical temperature decline resulting from increases in latitude and elevation. Subsequently, we conducted correlation analyses (Pearson correlation) between this environmental stress index and various reproductive parameters of the collected Twite populations (Figure 6).

The results showed that egg mass, egg volume, and nestling period exhibited positive correlations with environmental stress, whereas clutch size and incubation duration showed negative correlations with environmental stress. Naturally, environmental conditions across different habitats in the wild are highly complex and variable. Numerous factors, including latitude, elevation, temperature, precipitation, sunlight, monsoons, soil properties, and hydrology, interact with one another, collectively shaping local environments. Our analysis, however, only considered latitude and elevation and their associated effects on temperature as proxies for environmental stress. Consequently, this simplified representation inevitably overlooks other important ecological dimensions, which likely contributes to the generally weak statistical significance of the observed linear relationships. Nevertheless, the results still suggest, to some extent, that environmental stress—represented here by temperature—exhibits certain linear associations with various reproductive traits.

Overall, the findings of this study support the reproductive strategy commonly observed in birds inhabiting high-altitude, cold, and extreme environments: delayed breeding, a shortened breeding season, greater reproductive investment per offspring, and the production of fewer but larger eggs. Consistent with the findings of Lu and multiple other researchers, who all reached similar conclusions in their studies on the reproductive biology of high- and low-altitude populations of various bird species, including species *Turdus maximus* [42], *Trochalopteron Henrici* [43], *Onychostruthus taczanowskii* and *Pyrgilauda ruficollis* [44], *Luscinia phaenicuroides* [45], *Lanius tephronotus* [46], *Phoenicurus ochruros* [47], *Oenanthe isabellina* [48], *Petronia petronia* [49] and *Eremophila alpestris* [50].

In conclusion, compared to their low-altitude populations, the high-altitude birds produce smaller clutches but larger eggs, and have longer nestling periods, suggesting a life-history strategy adapted to harsh environments.

#### 4.2.2. Nest-Site Parameters Across Populations

Nest-site data were compiled from the Dangxiong region of Tibet [39], urban Lhasa in Tibet [51], northern England [52], and the present study, and are summarized below (Table 5).

Comparisons of the parameters showed that nest-site characteristics did not differ significantly among populations. An integrated analysis of nest-site data across habitats revealed that nest-site parameters of *Linaria flavirostris* populations distributed in different locations do not exhibit a consistent or systematic variation pattern, but are primarily influenced by local factors such as climate, vegetation composition, and anthropogenic activity.

Regarding nest height above ground, the populations ranked from highest to lowest were Lhasa, Dangxiong, Tianjun, and the UK population. This variation is attributed to differences in habitat characteristics. The study area for the Lhasa population was located within the urban district, where vegetation consisted mainly of tall, robust, and species-rich artificial forests. Data for the Dangxiong population were primarily collected from areas surrounding human settlements, where woodpiles near walls and roadside trees served as ideal nesting sites for the Twite. However, the present study was conducted in a wild environment with minimal anthropogenic disturbance; the vegetation consisted mostly of shrubs approximately one and a half meter in height, and only Sea buckthorn possessed the branched structure suitable for nesting. The UK population, however, predominantly nested in low shrubs or even on herbaceous plants.

In terms of nest mass, due to the developed livestock industry in the Tibetan region, the proportion of animal hair in the nests of the Dangxiong population was visibly higher. Although the Tianjun population also inhabits a pastoral area, its habitat is primarily riverbank scrub. The dense thorns of the Sea buckthorn make moving difficult and preclude grazing activities; consequently, the availability of animal hair for nesting material was limited.

## 5. Conclusions

This study on the breeding biology of the Twite (*Linaria flavirostris*) enriches the reproductive information on this species in a specific location, namely the northeastern QTP. It provides empirical evidence for the avian life-history strategy that “bird populations inhabiting higher altitudes produce larger and heavier eggs during breeding and invest more time and energy into fewer offspring.” Currently, the QTP is undergoing significant climate change. Most alpine bird species breeding there are facing habitat contraction and exhibit a trend of migrating to higher elevations. Therefore, clarifying the breeding information of alpine birds and characterizing their changing trends hold practical significance. The existing findings lay a foundation for understanding population dynamics and formulating conservation strategies for alpine birds, contributing to the protection of the Twite’s reproduction and the maintenance of its ecological functions on the QTP. Continuously improving and expanding reproductive data on birds in the Qinghai–Tibet Plateau is crucial for the understanding and conservation of this ecologically critical region.

## Figures and Tables

**Table 1:** Logistic growth curve fitting equations during the nestling period.

Item	Y = A/(1 + B × EXP(−K × t))	Y = A/2	t_A/2_	k_max_	R^2^
BM	Y = 12.449/(1 + 13.992 × EXP(−0.469 × t))	6.225	5.627	1.46	0.997
BL	Y = 105.635/(1 + 3.320 × EXP(−0.165 × t))	52.818	7.273	4.36	0.998
WL	Y = 55.561/(1 + 22.602 × EXP(−0.356 × t))	27.781	8.487	4.94	0.998
TL	Y = 38.593/(1 + 67.232 × EXP(−0.368 × t))	19.297	11.31	3.55	0.997
TSL	Y = 15.862/(1 + 5.295 × EXP(−0.407 × t))	7.931	3.527	1.61	0.993
BIL	Y = 7.073/(1 + 3.160 × EXP(−0.202 × t))	3.537	5.493	0.36	0.996

**Table 2:** Morphological traits of adults and differences between males and females.

Traits	Total	Male	Female	Z	*p*-Value
BL (mm)	132.0 ± 5.3 (n = 54)	133.1 ± 5.3 (n = 26)	130.9 ± 5.1 (n = 28)	−1.867	0.062
WL (mm)	74.1 ± 2.4 (n = 56)	75.0 ± 2.1 (n = 27)	73.2 ± 2.3 (n = 29)	−2.809	0.005
TL (mm)	66.2 ± 4.7 (n = 54)	67.9 ± 5.7 (n = 26)	64.7 ± 3.1 (n = 28)	−2.650	0.008
TSL (mm)	16.7 ± 1.2 (n = 56)	16.6 ± 1.0 (n = 27)	16.7 ± 1.3 (n = 29)	−0.025	0.980
BIL (mm)	7.8 ± 1.2 (n = 56)	7.8 ± 1.1 (n = 27)	7.8 ± 1.3 (n = 29)	−0.254	0.799
BIW (mm)	4.9 ± 0.5 (n = 30)	5.1 ± 0.4 (n = 16)	4.7 ± 0.6 (n = 14)	−1.754	0.079
BID (mm)	6.1 ± 0.6 (n = 30)	6.1 ± 0.6 (n = 16)	6.1 ± 0.6 (n = 14)	−0.230	0.818
HL (mm)	24.0 ± 0.8 (n = 56)	24.1 ± 0.7 (n = 27)	24.0 ± 0.9 (n = 29)	−0.427	0.670
HW (mm)	13.3 ± 0.6 (n = 30)	13.5 ± 0.6 (n = 16)	13.2 ± 0.6 (n = 14)	−0.813	0.416
HH (mm)	12.0 ± 0.7 (n = 30)	12.0 ± 0.6 (n = 16)	11.9 ± 0.8 (n = 14)	−0.209	0.834
BM (g)	12.9 ± 1.2 (n = 56)	12.7 ± 1.0 (n = 27)	13.0 ± 1.5 (n = 29)	−0.410	0.682

Note: *p*-values are based on the standardized test statistic (Z). The letter ‘n’ represents the sample size.

**Table 3:** Morphological traits of fledglings and adults.

Traits	Fledgling	Adult	Percentage
Body Mass (g)	12.3 ± 0.6	12.9 ± 1.2	95.35
Body Length (mm)	77.9 ± 4.2	132.0 ± 5.3	59.02
Wing Length (mm)	47.9 ± 2.2	74.1 ± 2.4	64.64
Tail Length (mm)	27.6 ± 3.6	66.2 ± 4.7	41.69
Tarsus Length (mm)	15.2 ± 0.5	16.7 ± 1.2	91.02
Bill Length (mm)	5.9 ± 0.5	7.8 ± 1.2	75.64

**Table 4:** Reproductive parameters of Twite breeding across different locations.

Parameters	Scotland	Pennines	S. Tibet	N. Tibet	C. Tibet	N. Qinghai	Present Study
Latitude (°N)	57.7	53.5	29.7	30.9	29.6	37.5	37.5
Altitude (m)	1200	500	3650	4300	3650	3200	3400
Breeding season (JD)	116–212	117–207	130–?	155–230	74–166	165–230	173–218
Breeding Duration (d)	96	90	-	75	92	65	45
Annual breeding attempts	2–3	1–2	-	-	1	-	1
Clutch size	5.2	5.5	4.0	4.2	4.1	4.5	4.7
Brood size at fledging	4.6	4.8	2.5	1.7	-	2.7	3.0
Egg volume (cm^3^) *	2.568	2.862	2.816	3.042	2.617	2.635	2.786
Fresh egg mass (g)	1.4	1.5	-	1.6	1.4	1.3	1.5
Incubation period (d)	11.3	-	-	12.2	13.9	13.0	11.5
Nestling period (d)	15.5	-	-	14.7	11.3	14.0	12.4

* The volume is calculated as the product of the Length and the square of the Breadth, differing from method above.

**Table 5:** Nest parameters of Twite breeding in various habitats.

*Parameters*	*N. England*	*DangXiong*	*Urban Lhasa*	*Present Study*
*Height above the ground (cm)*	*0–90.0*	*0–300.0*	*289.3 ± 149.6*	*127.2 ± 31.5*
*Height of the cup (cm)*	*5*	*–*	*7.7 ± 1.2*	*6.5 ± 0.7*
*Depth of the cup (cm)*	*3*	*4.2 ± 0.7*	*3.8 ± 0.8*	*3.7 ± 0.6*
*Inner-diameter (cm)*	*4*	*6.2 ± 0.8*	*4.9 ± 0.9*	*5.8 ± 0.8*
*Out-diameter (cm)*	*9*	*9.3 ± 1.9*	*9.0 ± 1.6*	*9.9 ± 0.8*
*Dry mass (g)*	*–*	*33.3 ± 22.0*	*19.3 ± 6.7*	*17.5 ± 5.3*

## Data Availability

Further information on the data included in this study is available from authors upon reasonable request.

## References

[1] Zhang X. (2014). Study on Breeding Biology of Brown Shrike (*Lanius cristatus*) in Heilongjiang Maoershan Mountain of Northeast China. Master’s Thesis.

[2] Li H. (2021). The Breeding Ecology of Snow Partridge (*Lerwa lerwa*). Master’s Thesis.

[3] Liu N., Bao X., Liao J. (2013). The Classification and Distribution of the Birds in Qingzang Plateau.

[4] Dunning J. (2018). The Spatial and Molecular Ecology of Twite *Linaria flavirostris*. Master’s Thesis.

[5] Perrins C.M. (1970). The timing of birds’ breeding seasons. Ibis.

[6] Visser M.E., Both C. (2005). Shifts in phenology due to global climate change: The need for a yardstick. Proc. R. Soc. B.

[7] Martin T.E. (1995). Avian life history evolution in relation to nest sites, predation, and climate. Ecol. Monogr..

[8] Jetz W., Sekercioglu C.H., Bhning-Gaese K. (2008). The worldwide variation in avian clutch size across species and space. PLoS Biol..

[9] Johnson L.S., Brubaker J.L., Ostlind E., Balenger S.L. (2007). Effect of altitude on male parental expenditure in Mountain Bluebirds (*Sialia currucoides*): Are higher-altitude males more attentive fathers?. J. Ornithol..

[10] Badyaev A.V., Ghalambor C.K. (2008). Evolution of life histories along elevational gradients: Trade-off between parental care and fecundity. Ecology.

[11] MacKinnon J., Phillips K., He F. (2000). A Field Guide to the Birds of China.

[12] Zheng G., Zhang H. (2023). Checklist of the Birds of China: Taxonomy and Distribution.

[13] Editorial Committee of Fauna Sinica, Chinese Academy of Sciences (1978). Fauna Sinica: Aves.

[14] Clement P., del Hoyo J., Elliott A., Sargatal J., Christie D.A., de Juana E. (2020). Twite (*Linaria flavirostris*), version 1.0. Birds of the World.

[15] National Forestry and Grassland Administration of China (2023). List of Terrestrial Wildlife with Important Ecological, Scientific, and Social Values.

[16] Zhao L., Li L., Zhang X. (2002). Effects of hatching behavior on offspring quality in two species passerines. Zool. Res..

[17] Zhao L., Liu Z., Zhang X., Yi X., Li M. (2003). Feeding nestling in Twite *Acanthis flavirostris* at the Haibei alpine meadow, Qinhai. Zool. Res..

[18] Raine A.F., Sowter D.J., Brown A.F., Sutherland W.J. (2006). Natal philopatry and local movement patterns of Twite *Carduelis flavirostris*. Ringing Migr..

[19] Raine A.F., Brown A.F., Amano T., Sutherland J.W. (2009). Assessing population changes from disparate data sources: The decline of the Twite *Carduelis flavirostris* in England. Bird Conserv. Int..

[20] Corse C.J., Clark H., Duncan R., Mainwood T., Patterson D., Wells L., Adam R.G., Ribbands J.B. (2001). Movements of Twite *Carduelis flavirostris* in northern Scotland. Ringing Migr..

[21] Wilkinson N.I., Eaton M.A., Colhoun K., Drewitt A.L. (2018). The population status of breeding Twite *Linaria flavirostris* in the UK in 2013. Bird Study.

[22] Dunning J., Finch T., Davison A., Durrant K.L. (2020). Population-specific migratory strategies of Twite *Linaria flavirostris* in Western Europe. Ibis.

[23] Gkourtsouli-Antoniadou I., Ewing S.R., Hudson G., Pearson M.A., Schroeder J., Welch P.E., Wilkinson N.I., Dunning J. (2023). Age-specific survival in an English Twite *Linaria flavirostris* population. Bird Study.

[24] Dong Z., He P., Song G., Zhang S., He X., Wu Y. (2021). Collection and preservation of research materials (digital recording, tissue samples) and methods of taxidermy related to wild birds. Bio-protocal.

[25] Narushin V.G., Romanov M.N., Griffin D.K. (2025). Pear-Shaped Eggs Evolved to Maximize the Surface Area-to-Volume Ratio, Increase Metabolism, and Shorten Incubation Time in Birds. Integr. Zool..

[26] Hoyt D.F. (1979). Practical methods of estimating volume and fresh weight of bird eggs. Auk.

[27] Zhang Z., Li Q., Cai Y., Yang C. (2025). Revealing the key signals in nestling begging behavior perceived by parent birds during parent–offspring conflict. Integr. Zool..

[28] Mayfield H. (1961). Nesting success calculated from exposure. Wilson Bull.

[29] Dinsmore S.J., White G.C., Knopf F.L. (2002). Advanced techniques for modeling avian nest survival. Ecology.

[30] Ricklefs R.E. (1968). Patterns of growth in birds. Ibis.

[31] Zach R. (1982). Nestling house wrens: Weight and feather growth. Can. J. Zool..

[32] Tjørve K., Tjørve E. (2010). Shapes and functions of bird-growth models: How to characterise chick postnatal growth. Zoology.

[33] Hossein-Zadeh N.G. (2025). Comparison of nonlinear models for describing the growth curve of pekin ducks. Vet. Med. Sci..

[34] Nice M.M. (1957). Nesting success in altricial birds. Auk.

[35] Bailey R.L., Larson L. (2024). NestWatch: An open-access, long-term data set on avian reproductive success. Ecology.

[36] Xu Y., Zhu H., Chen X., Wang J., Wang Y. (2025). Multidimensional nestedness patterns and underlying processes of bird assemblages in the Zhoushan Archipelago, China. Avian Res..

[37] Martin T.E. (1993). Nest predation and nest sites: New perspectives on old patterns. BioScience.

[38] Brown A.F., Crick H.Q.P., Stillman R.A. (1995). The distribution, numbers and breeding ecology of Twite *Acanthis flavirostris* in the south Pennines of England. Bird Study.

[39] Lu X., Guo Y., Liang J., Ma X., Zhang L. (2011). Breeding ecology of the Twite *Carduelis flavirostris* in northern Tibet. Ornis Fenn..

[40] Zhou S. (2016). Habitat Selection and Breeding Ecology Research of Twite in Lhasa River Valley. Master’s Thesis.

[41] Zhao L., Zhang X., Liu Z. (2005). Breeding ecology of passerine birds in alpine meadows of northern Qinghai. Zool. Res..

[42] Lu X. (2005). Reproductive ecology of blackbirds (*Turdus merula maximus*) in a high-altitude location, Tibet. J. Ornithol..

[43] Lu X., Gong G., Zeng X. (2008). Reproductive ecology of Brown-cheeked Laughing Thrushes (*Garrulax henrici*) in Tibet. J. Field Ornithol..

[44] Lu X., Ke D., Zeng X., Yu T. (2009). Reproductive ecology of two sympatric Tibetan snowfinch species at the edge of their altitudinal range: Response to more stressful environments. J. Arid Environ..

[45] Lu X., Wang C., Yu T. (2010). Nesting ecology of the Grey-backed Shrike (*Lanius tephronotus*) in south Tibet. Wilson J. Ornithol..

[46] Lu X., Yu T., Liang W., Yang C. (2010). Comparative breeding ecology of two White-bellied Redstart populations at different altitudes. J. Field Ornithol..

[47] Lu X., Ke D., Guo Y., Tang S., Zhang L., Wang C. (2011). Breeding ecology of the Black Redstart *phoenicurus ochruros* at a Tibetan site, with special reference to cooperative breeding. Ardea.

[48] Li S., Lu X. (2012). Breeding biology of Rock Sparrows *Petronia petronia* in the Tibetan Plateau, with special reference to life history variation across altitudes. Acta Ornithol..

[49] Li S., Lu X. (2012). Reproductive ecology of Isabelline Wheatears at the extreme of their altitude distribution. Ardeola.

[50] Li S., Guo C., Peng W. (2016). Breeding patterns of Asian Horned Larks (*Eremophila alpestris nigrifrons*) on the Tibet Plateau. Wilson J. Ornithol..

[51] Zhou S., Pu-Bu, Tsering-Dorge (2017). Preliminary report on nest-site selection by *Carduelis flavirostris* in urban area of Lhasa. Sichuan J. Zool..

[52] del Hoyo J., Elliott A., Christie D.A. (1992). Handbook of the Birds of the World.

